# Transcriptome Analysis of the Oriental Fruit Fly (*Bactrocera dorsalis*)

**DOI:** 10.1371/journal.pone.0029127

**Published:** 2011-12-15

**Authors:** Guang-Mao Shen, Wei Dou, Jin-Zhi Niu, Hong-Bo Jiang, Wen-Jia Yang, Fu-Xian Jia, Fei Hu, Lin Cong, Jin-Jun Wang

**Affiliations:** Key Laboratory of Entomology and Pest Control Engineering, College of Plant Protection, Southwest University, Chongqing, People's Republic of China; University of Miami, United States of America

## Abstract

**Background:**

The oriental fruit fly, *Bactrocera dorsalis* (Hendel), is one of the most economically important pests in the world, causing serious damage to fruit production. However, lack of genetic information on this organism is an obstacle to understanding the mechanisms behind its development and its ability to resist insecticides. Analysis of the *B. dorsalis* transcriptome and its expression profile data is essential to extending the genetic information resources on this species, providing a shortcut that will support studies on *B. dorsalis*.

**Methodology/Principal Findings:**

We performed *de novo* assembly of a transcriptome using short read sequencing technology (Illumina). The results generated 484,628 contigs, 70,640 scaffolds, and 49,804 unigenes. Of those unigenes, 27,455 (55.13%) matched known proteins in the NCBI database, as determined by BLAST search. Clusters of orthologous groups (COG), gene orthology (GO), and the Kyoto Encyclopedia of Genes and Genomes (KEGG) annotations were performed to better understand the functions of these unigenes. Genes related to insecticide resistance were analyzed in additional detail. Digital gene expression (DGE) libraries showed differences in gene expression profiles at different developmental stages (eggs, third-instar larvae, pupae, and adults). To confirm the DGE results, the expression profiles of six randomly selected genes were analyzed.

**Conclusion/Significance:**

This transcriptome greatly improves our genetic understanding of *B. dorsalis* and makes a huge number of gene sequences available for further study, including both genes of known importance and genes of unknown function. The DGE data provide comprehensive insight into gene expression profiles at different developmental stages. This facilitates the study of the role of each gene in the developmental process and in insecticide resistance.

## Introduction

The oriental fruit fly, *Bactrocera dorsalis* (Hendel) belongs to the *B. dorsalis* complex. This pest has gained international significance in that it is a highly invasive species that has greatly expanded its geographic distribution over the last century. This insect has been found in Asia and the Pacific islands, where it causes severe losses to many commercially important tropical and subtropical crops, especially fruits. Some entomologists and quarantine biologists consider *B. dorsalis* to be one of the most important pest species in world agriculture [1]. The female oviposits inside the fruit, where the larvae feed until pupation. This often causes fruit damage and fruit drop [2]. *B. dorsalis* is polyphagous as well as highly invasive, so many countries impose strict quarantine restrictions to prevent its expansion to new host plants and geographic areas. These restrictions limit the world trade in agricultural commodities [3], [4]. In fine, because of its invasive ability, wide geographic distribution and host range, pest status, and impact on market access, *B. dorsalis* is considered a major threat to global agriculture [5].

Over the past few decades, a great deal of research has been conducted on the basic ecological and biological characteristics of *B. dorsalis*, but the mechanisms behind molecular regulation in this species remain poorly understood [6], [7]. In recent years, genes related to development and stress tolerance have been studied as potential targets for effective management of this pest [8], [9]. The studies on the mechanism behind organophosphate insecticide resistance in *B. dorsalis* are an excellent example of the utility of this research strategy [10], [11]. Such molecular techniques can also yield insights into basic biology and ecology [12], [13], [14].

Even with the current achievements on molecular regulation of *B. dorsalis*, a comprehensive view of this species has yet to form, largely due to the lack of genomic information. As of May 28, 2011, only 881 *B. dorsalis* nucleotide sequences and 615 protein sequences have been deposited in the NCBI database. These data are far from sufficient, and most of the important genes related to development (*e.g.*, juvenile hormone, eclosion hormone) and insecticide resistance (*e.g.*, P450, GSTs) are still unknown. Gene sequences are difficult to fully characterize using traditional biochemical methods, and PCR combined with RACE is a lengthy, sometimes inefficient process [15]. The emergence of next-generation high-throughput DNA sequencing techniques has provided an opportunity for researchers to quickly and efficiently obtain massive quantities of genetic data [16]. The Illumina technique for transcriptome analysis has been used to investigate human diseases, as well as mammals, plants, and insects [17]–[22]. In insects, Illumina transcriptome analysis has been shown to be a reliable and precise way to study genomic characteristics, including development, insecticide targets, detoxifying enzymes, metabolism and immune response, and tissue specificity [15], [23]–[29]. This technique has not yet been applied to *B. dorsalis*, but we expect that a transcriptome analysis will greatly improve our understanding of *B. dorsalis* at the molecular level.

In this study, we used short read sequencing technology (Illumina) for *de novo* transcriptome analysis. We constructed a library covering four life stages of *B. dorsalis*, eggs, third-instar larvae, pupae, and adults. Nearly 27 million reads of a total of 2.4 billion nucleotides (nt) were assembled into 49,804 unigenes. Of those unigenes, 27,455 (55.13%) matched known proteins in a BLAST search of the NCBI database. Matches included a number of genes related to insecticide resistance. We also compiled four digital gene expression (DGE) libraries to investigate the expression profiles of genes at different developmental stages (eggs, third-instar larvae, pupae, and adults). These assembled, annotated transcriptome sequences and gene expression profiles extend the genomic resources available for researchers studying *B. dorsalis* and may provide a fast approach to identifying genes involved in development and insecticide resistance.

## Results

### Sequencing and sequence assembly

A library (SRA submission number: SRA040301.1) of four developmental stages (eggs, third-instar larvae, pupae, and adults) was constructed by Illumina sequencing in a single run which generated 26,666,670 total reads (2×90 bp) and 2,400,000,300 nucleotides (nt) (Table 1). These short reads were assembled into 484,628 contigs with a mean length of 137 bp. These contigs were further connected into 70,640 scaffolds using the SOAPdenovo program with a mean length of 358 bp. Finally, after gap filling of scaffolds using paired-end reads from the transcriptome sequencing data, we obtained 49,804 unigenes. The mean size of these unigenes was 456 bp and lengths ranged from 150 to 7,797 bp. Of these unigenes, 4,404 were larger than 1,000 bp (Figure S1).

**Table 1:** Summary of the transcriptome.

Total reads	26,666,670
Total nucleotides (nt)	2,400,000,300
Total number of contigs	484,628
Total number of scaffolds	70,640
Total number of unigenes	49,804
Sequences with E-value<10^−5^	27,455

### Annotation of predicted proteins

**Figure 1:**
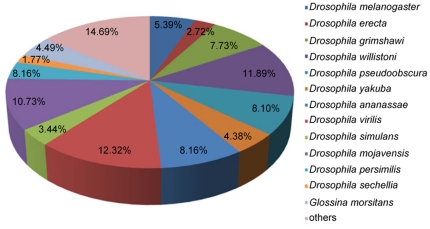
Species distribution of the BLASTX matches of the transcriptome unigenes. This figure shows the species distribution of unigene BLASTX matches against the nr protein database (cutoff value E<10^−5^) and the proportions for each species.

Unigene sequences were annotated by searching the non-redundant (nr) NCBI protein database using BLASTX with a cut-off E-value of 10^−5^. A total of 27,455 distinct sequences (55.13% of unigenes) matched known genes (Table S1). The majority of sequences (79.47%) had strong homology with *Drosophila* (Figure 1). Of these, 12.32% of the unigenes were best matched to sequences from *D. virilis*, followed by *D. willistoni* (11.89%), *D. mojavensis* (10.73%), and other species within *Drosophila*. The other sequences, which made up 20.53% of the total, had hits with other insect species, such as *Tribolium castaneum* (0.59%), *Apis mellifera* (0.26%), and *Bombyx mori* (0.18%). Compared to other species within Diptera, 4.49% of sequences matched sequences from *Glossina morsitans morsitans*, 0.86% from *Aedes aegypti*, and 0.16 from *Musca domestica*.

### Unigene function annotation

**Figure 2:**
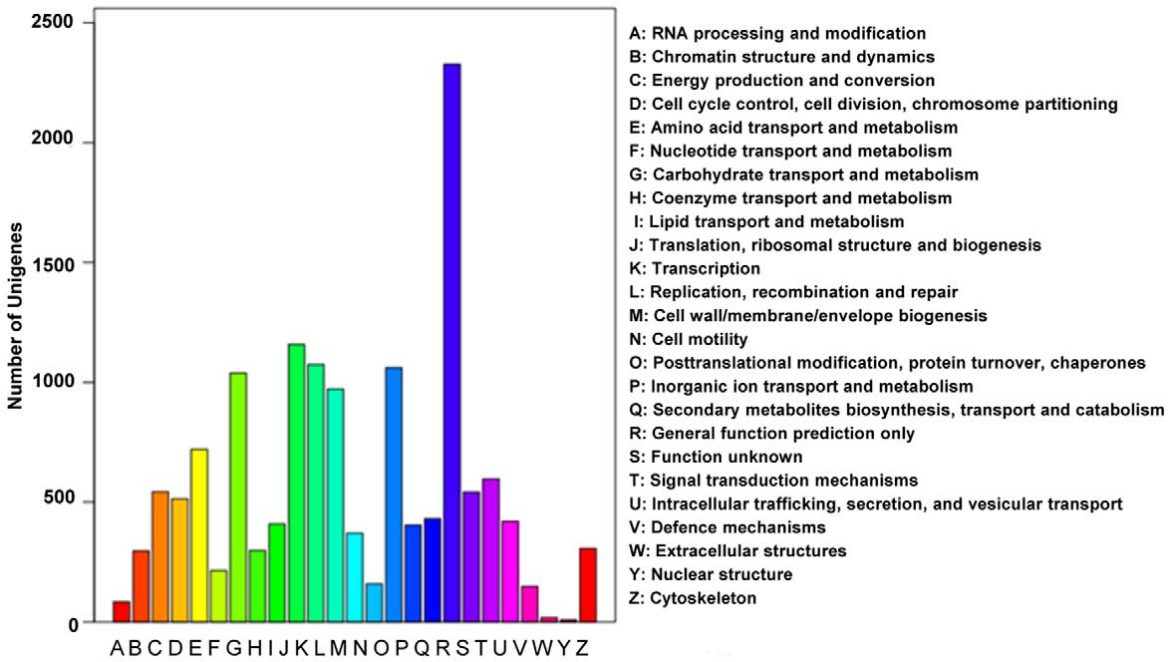
Classification of the clusters of orthologous groups (COG) for the transcriptome of *Bactrocera dorsalis*. 14,108 unigenes (51.39% of the total) were annotated and divided into 26 specific categories.

Assignments of clusters of orthologous groups (COG) were used to predict and classify possible functions of the unigenes. Based on sequence homology, 14,108 unigenes (51.39%) were annotated and divided into 26 specific categories (Figure 2). The general function category, which contained 2,327 unigenes (16.49%), was the largest, followed by translation, ribosomal structure, and biogenesis (1,158, 8.21%), transcription (1,074, 7.61%), post-translational modification, protein turnover, and chaperones (1,060, 7.52%), and carbohydrate transport and metabolism (1,039, 7.36%). Only eight unigenes (0.057%) belonged to nuclear structure, which was the smallest group.

**Figure 3:**
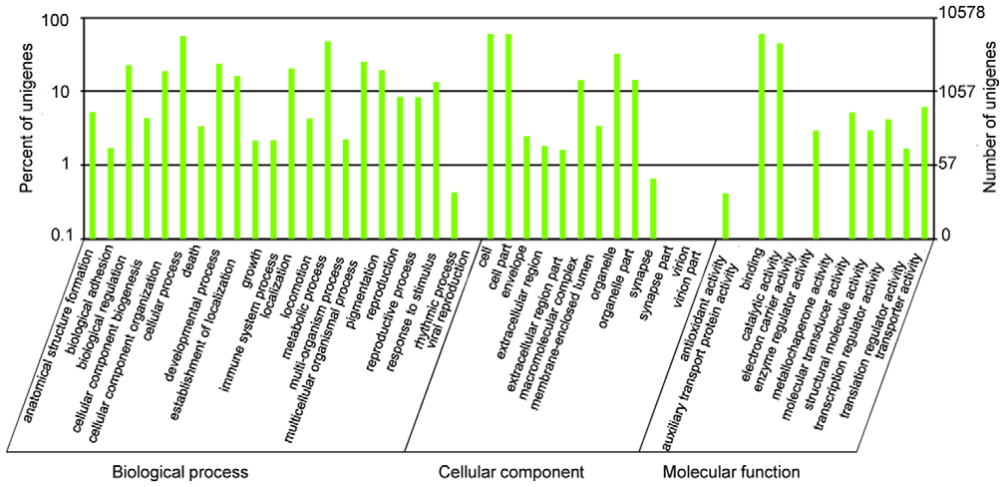
Classification of the gene ontology (GO) for the transcriptome of *Bactrocera dorsalis*. 10,578 unigenes (38.53% of the total) were categorized into 47 function groups.

For gene ontology (GO) analysis, unigenes were divided into three ontologies: molecular function, cellular component, and biological process. We categorized 10,578 unigenes (38.53% of total) into 47 function groups. Binding and cell component were the two largest groups, containing 6,334 and 6,319 unigenes, respectively. Only one unigene each was predicted to act in the functional groups metallochaperone activity and electron carrier activity (Figure 3).

### Unigene metabolic pathway analysis

The unigene metabolic pathway analysis was conducted using the Kyoto Encyclopedia of Genes and Genomes (KEGG) annotation system. We mapped 16,141 unigenes (58.79%) to 219 KEGG pathways. Metabolic pathways contained 2,971 unigenes (18.41%) and was significantly larger than other pathways, such as pathways in cancer (4.29%), focal adhesion (3.38%), and spliceosome (3.15%) (Table S2).

### Transcripts encoding specific genes of insecticide detoxification and target enzymes

To obtain interested unique sequences related to insecticide resistance, unigenes detected in the library were manually curated by removing redundant and overly short sequences. Glutathione *S*-transferases (GSTs), carboxylesterases (CarEs), and cytochrome P450 (P450) were identified as three major representative detoxifying enzymes. They were further divided into different classes. A number of sequences encoding insecticide targets were also indentified, such as acetylcholinesterase (AChE) (*eg.* unigene number: 10466), the γ-aminobutyric acid receptor (GABA) (*eg.* unigene number: 12690), sodium channel (*eg.* unigene number: 49368), and nicotinic acetylcholine receptor subunits (nAChRs). Specific sequence information for these unigenes is shown in Table S3.

**Figure 4:**
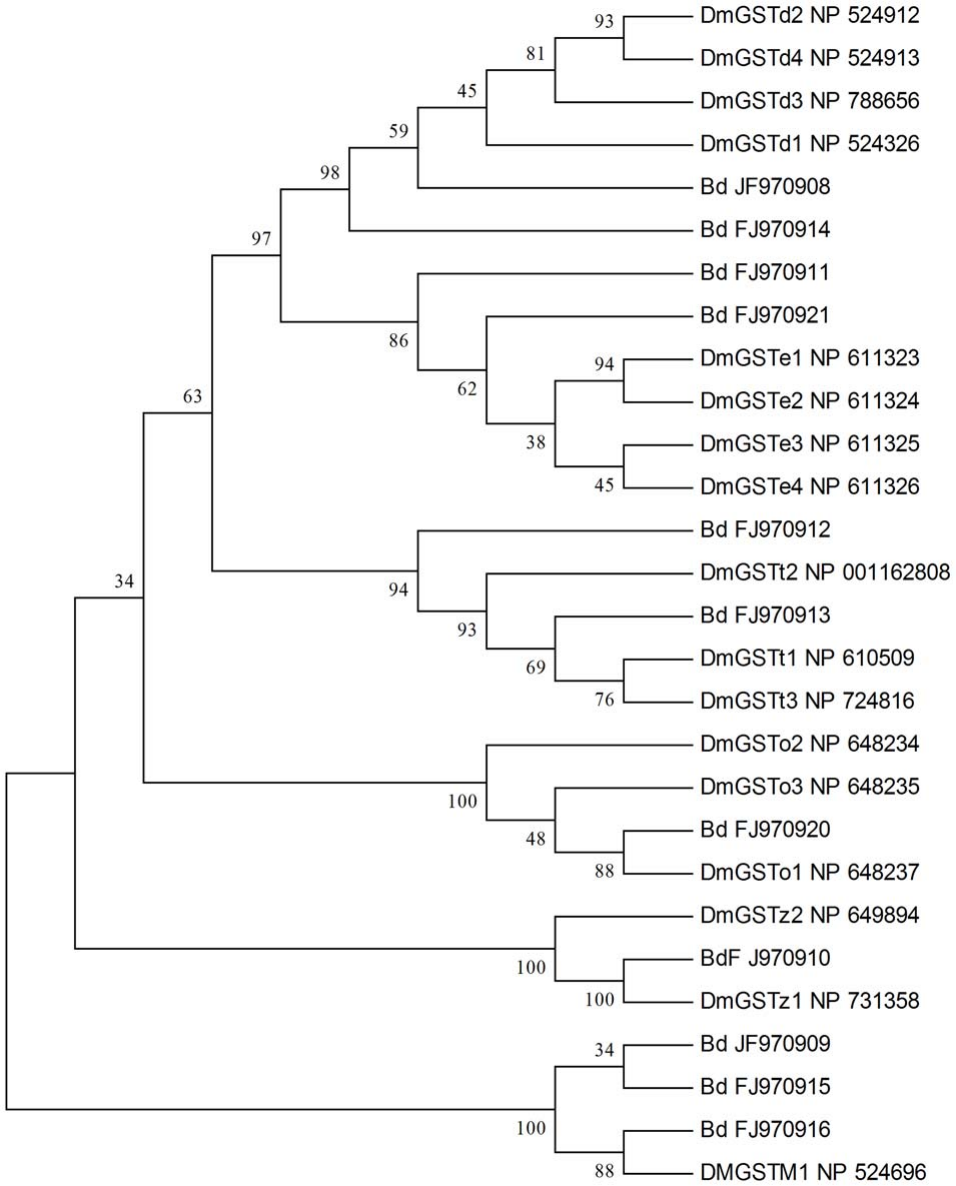
Neighbor-joining phylogenetic analysis of the glutathione S-transferases from *Bactrocera dorsalis* (Bd) and *Drosophila melanogaster* (Dm).

Of the 48 GSTs-related sequences in the transcriptome data, 14 unique gene sequences with an average length of 371 bp encoding specific GSTs genes were identified (JF970908–JF970921). Among these, 11 genes were classified into six classes including delta, epsilon, omega, theta, zeta, and microsomal class by phylogenetic analysis with GST genes from *D. melanogaster* (Figure 4). Another three GSTs genes were assigned to epsilon (JF970917), omega (JF970919), and zeta (JF970918) classes by their closest BLAST hit in the nr database.

**Figure 5:**
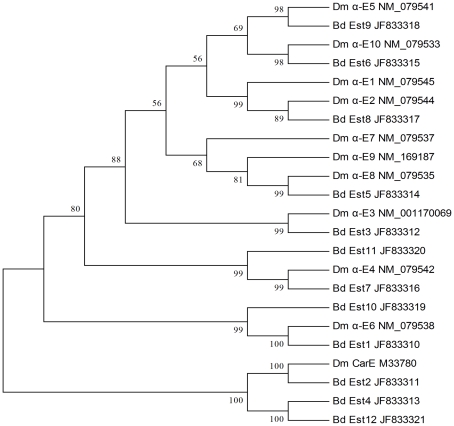
Neighbor-joining phylogenetic analysis of the carboxylesterases from *Bactrocera dorsalis* (Bd) and *Drosophila melanogaster* (Dm).

Twelve sequences encoding CarEs, average length of 373 bp, were identified from 150 unigenes encoding esterases (JF833310–JF83321). Phylogenetic analysis with genes from *D. melanogaster* was carried out, and 9 sequences showed high homology with α-esterase, an important component of CarEs (Figure 5).

**Figure 6:**
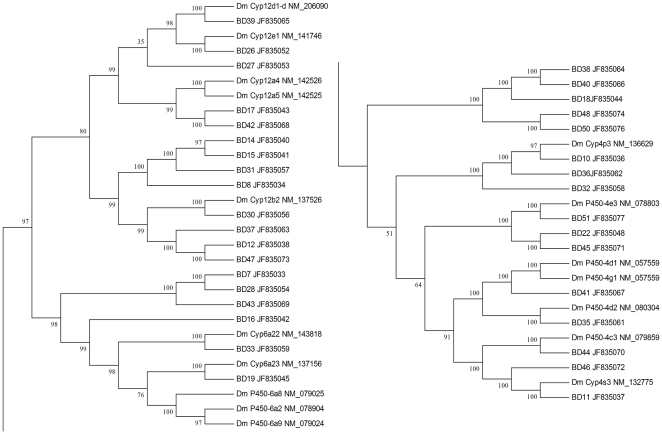
Neighbor-joining phylogenetic analysis of cytochrome P450 from *Bactrocera dorsalis* (Bd) and *Drosophila melanogaster* (Dm).

A total of 246 P450-related sequences were obtained from the transcriptome data. Of these, 51 sequences with an average length of 575 bp were identified to encode specific P450 genes (JF835027–JF835077). These identifications were based on the best match in nr database according to the BLAST results and, when possible, on phylogenetic analysis with P450 genes from *D. melanogaster*. Most of these genes were classified into CYP4, CYP6, and CYP12 families (Figure 6).

**Figure 7:**
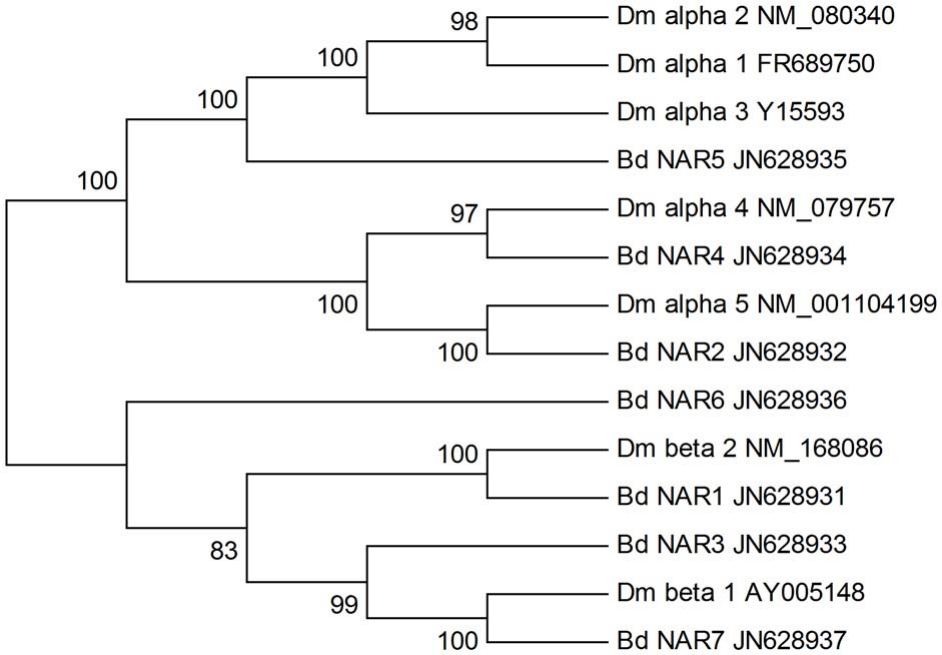
Neighbor-joining phylogenetic analysis of the nicotinic acetylcholine receptor from *Bactrocera dorsalis* (Bd) and *Drosophila melanogaster* (Dm).

Seven sequences encoding nAChR genes were deposited into GenBank (JN628931–JN628937). Based on the phylogenetic analysis, four were assigned to the alpha subunit group and the other three were assigned to the beta subunit group (Figure 7). Two sequences that were shorter than the 200 bp limit showed high homology with the nAChR alpha subunit of *Culex quinquefasciatus* and *Anopheles gambiae*, according to the BLAST results in nr database.

### Digital gene expression (DGE) library sequencing

Based on the transcriptome sequence data, four DGE libraries were constructed to identify the unigene expression profiles of the different developmental stages (accession numbers: SRA043792.1 for eggs, SRA043786.1 for third-instar larvae, SRA043785.1 for pupae, and SRA043783.1 for adults). After removing low-quality reads, each library generated approximately six million clean reads. Among these clean reads, 1.3–2.3 million (21.60–39.40%), were mapped to unigenes in four libraries (Table 2). The percentage of clean reads ranged from 98.92% to 99.12%, reflecting the high quality of the sequencing (Figure S2). More than 19% of genes were covered between 90–100% in each developmental stage. Fewer than 2% of the genes were covered by 0–10%. The distribution of genes with coverage above 50% was 75% in eggs, 60% in larvae, 77% in pupae, and 63% in adults (Figure S3).

**Table 2:** Alignment statistics of the RNA-Seq analysis.

Summary	Eggs	Larvae	Pupae	Adults
Total reads	5,948,945	5,994,823	6,128,403	5,982,870
Total base pairs	291,498,305	293,746,327	300,291,747	293,160,630
Total mapped reads	2,343,700	1,295,060	2,233,481	1,803,245
Perfect match	1,876,283	967,809	1,662,186	1,279,540
≤2 bp mismatch	467,417	327,251	571,295	523,750
Unique match	2,343,591	1,294,684	2,233,077	1,803,017
Multi-position match	109	376	404	228
Total unmapped reads	3,605,245	4,699,763	3,894,922	4,179,625

### Comparison of gene expression profile among the different developmental stages

**Figure 8:**
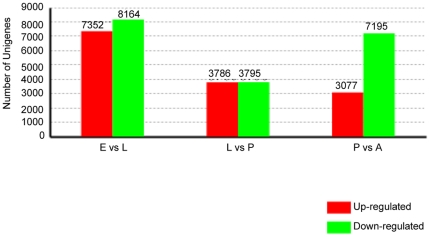
Summary differently expressed genes in each pairwise comparison. E vs. L: eggs and larvae; L vs. P: larvae and pupae; P vs. A: pupae and adults.

The four developmental stages were evaluated in three pairwise comparisons: eggs vs. third-instar larvae (E vs. L), third-instar larvae vs. pupae (L vs. P), and pupae vs. adults (P vs. A). Genes found to have significant differences in expression were identified in each comparison (Figure 8). The results suggested that the expression of 15,516 genes was significantly different in eggs and third-instar larvae. Of these genes 7,352 were up-regulated and 8,164 were down-regulated in the E vs. L comparison. Only two genes among the top ten up-regulated genes could be matched to any known function in GenBank. They were predicted to be anoxia (*D. yakuba*, AAQ09892.1) and larval serum protein 1 beta (*D. melanogaster*, NP_476624.1). Two genes matching the gene encoding cyclin from *D. erecta* (XP_001976294.1) and blastoderm-specific gene from *D. melanogaster* (NP_523472.2) were found among the top ten down-regulated genes. The other 14 genes among the top 20 differently expressed genes encoded proteins of unknown function (Table S4). In the comparison of third-instar larvae and pupae, the expression profiles of 7,581 genes had changed. There were 3,786 genes up-regulated in pupae and 3,795 genes that were down-regulated. Among the top ten up-regulated genes, one matched the gene encoding the hemocyanin protein of *D. pseudoobscura* (XP_001353545.1). Although another gene showed homology with a gene from *D. persimilis* (XP_002025718.1), the function of the protein encoded by this gene is not known. Among the top ten down-regulated genes, three genes matched the gene encoding the yippee interacting protein of *D. melanogaster* (AAF27820.1), the chitin binding protein of *D. willistoni* (XP_002061900.1), and the hemolymph juvenile hormone binding protein (JHBP) of *D. ananassae* (XP_001964613.1). The other two showed homology with two unknown proteins from *A. gambiae* (XP_314057.4) and *D. erecta* (XP_001973287.1) (Table S5). When comparing pupae and adults, 3,077 genes were up-regulated in adults and 7,195 genes were down-regulated. One of the top ten up-regulated genes showed homology with a gene encoding flightin-like protein in *Acyrthosiphon pisum* (XP_001944120.1). The other two matched genes encoding unknown proteins in *D. ananassae* (XP_001962144.1) and *A. gambiae* (XP_001688814.1). The proteins encoded by three genes among the top ten down-regulated matched the insect cuticle protein of *D. ananassae* (XP_001965585.1), the arylphorin receptor of *Calliphora vicina* (CAA55707.1), and serine protease inhibitors of *D. grimshawi* (XP_001983639.1). One gene showed homology with a gene from *D. persimilis* (XP_002022926.1), but the specific function of this gene remains unknown (Table S6).

Based on the GO classification, differently expressed genes were characterized into three groups: biological process, cellular component, and molecular function. The results of each comparison showed high accordance with genes related to biological processes mainly concentrated in the cellular process category (2,703 genes in E vs. L, 1,206 genes in L vs. P, 1,974 genes in P vs. A) and metabolic process category (2,176 genes in E vs. L, 1,101 genes in L vs. P, 1,569 genes in P vs. A). In the comparison of egg and larvae, 2,205 and 1,170 cellular component genes were involved in the intracellular and organelle categories, respectively. In the other two comparisons, the numbers were 1,361 and 933 (L vs. P), and 2,118 and 1,541 (P vs. A), respectively. Finally, 2,794 (E vs. L), 1,269 (L vs. P) and 1,998 (P vs. A) genes were involved in binding. In the pathway analysis, the most differently expressed genes were involved in metabolic pathways. The number of differently expressed genes was significantly higher than in the other pathways (Tables S7, S8, and S9).

### Validation of gene expression profile

**Figure 9:**
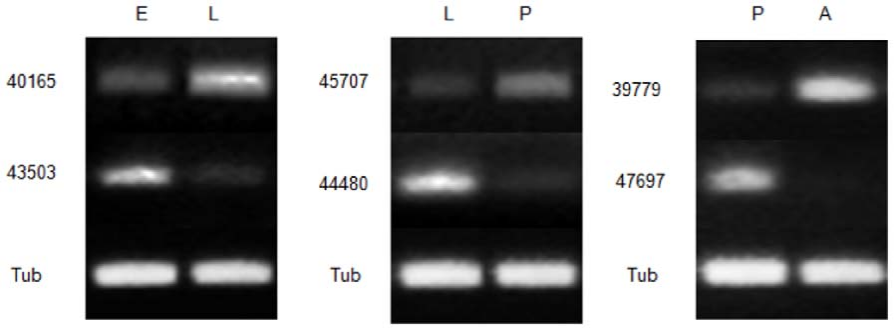
Validation of gene expression by semi-quantitative RT-PCR. Multiple gel images are here shown as a composite figure. E: eggs; L: larvae; P: pupae; A: adults.

To confirm the gene expression profiles, six genes were randomly picked from among the top ten up-regulated and down-regulated of each pairwise comparison. The 1% agarose gel electrophoresis of PCR products with α-tubulin gene as an internal control showed that the expression profiles of three genes (unigene number: 40165 in E vs. L, 45707 in L vs. P, and 39779 in P vs. A) were significantly higher and those of the others (unigene number: 45303 in E vs. L, 44480 in L vs. P and 47697 in P vs. A) were lower than the comparative stage in each group (Figure 9). The results agreed perfectly with the DGE analysis, suggesting that both sets of results are reliable.

## Discussion

The oriental fruit fly, *B. dorsalis* (Hendel), is a biologically interesting and economically relevant insect in the order Diptera. It is a member of the most important group of insect pests, causing severe damage to fruit products globally [2]. Recent studies have focused on its biology and ecology, but a lack of genetic information is still a barrier to further understanding this species.

After Illumina sequencing of *B. dorsalis*, 49,804 unigenes were detected; 55.13% of them (27,455) were found to have significant homology to functional genes encoding specific proteins using the BLASTX analysis in GenBank. Homology analysis of the unigenes demonstrated that 79.47% showed the greatest similarity to *Drosophila*; 11 species of *Drosophila* had a best match percentage greater than 2% (Figure 1). Actually, as a member of Diptera, *B. dorsalis* has a much closer relationship with *Drosophila* than with Homopteran species (*Laodelphax striatellus*, *Nilaparvata lugens* or *Bemisia tabaci*) [23], [24], [30]. *Drosophila* is, of course, an incredibly important model organism for both insect biology and for the life sciences in general. *Drosophila* genomics have been well studied and are an important reference for genetic research into other organisms [31]. The distribution was in accordance with the results of transcriptome analysis of another dipteran species, *Glossina morsitans morsitans*, of which 81% genes were most closely related to those of *Drosophila* species [28]. We noted that only 41 unigene sequences (0.15%) matched recorded sequences of *B. dorsalis*. This suggests that the genes that we identified have made a meaningful contribution to the body of knowledge of *B. dorsalis*.

According to the blast results in GenBank, only 881 nucleotide sequences of *B. dorsalis* had been previously submitted (prior to May 28, 2011). One of the purposes of this is to find an efficient way to control this pest. In recent years, in China, geographically widespread populations of *B. dorsalis* have developed high levels of resistance to commonly used insecticides, such as trichlorphon, β-cypermethrin, and avermectin [32]. However, the molecular mechanisms of resistance are still unknown and the main obstacle to further research is the limited amount of genetic information. To assist research on insecticide resistance, we surveyed our transcriptome database and identified the most important enzymes related to the metabolism of insecticides or genes encoding proteins that are the targets of insecticides.

The P450s are a major family of enzymes involved in detoxification and metabolism [33]. Before our study, only seven P450 sequences of *B. dorsalis* were available in GenBank. In this study, 51 additional unique sequences encoding P450 genes were selected and submitted. These genes belong to several families; most are members of the CYP4, CYP6, and CYP12 families, according to phylogenetic analysis and BLAST results.

GSTs play an important role in phase II detoxification of the hydrophobic toxic compounds found in insecticides. They are thought to be mainly involved in the detoxification of organophosphates, pyrethroids, and organochlorines [34]–[36]. In *D. melanogaster*, 37 GSTs genes have been identified. However, none had been reported for *B. dorsalis*. We characterized 14 GSTs genes of *B. dorsalis* from the transcriptome database. This should provide valuable new information for future studies. GSTs are a diverse superfamily. We classified 11 GSTs genes belonging to five families; three in epsilon (JF970911, JF970917, JF970921), two in delta (JF970914, JF970908), two in omega (JF970919, JF970920), two in zeta (JF970910, JF970918), and two in theta (JF970912, JF970913). The other three were considered to be microsomal (JF970909, JF970915, JF970916).

Physiological functions of CarEs include many aspects, including degradation of neurotransmitters, metabolism of specific hormones and pheromones, detoxification, defense, and behavior [37]. In insects, mutations occurring in CarEs genes could potentially increase the rate of insecticide hydrolysis, such as that of organophosphates, or it could decrease activity towards generic substrates, such as naphthyl acetate [38]. Although many point mutations related to insecticide resistance have been found in insects no CarEs genetic information had been reported for *B. dorsalis* [39]. In the transcriptome database, 12 CarEs gene sequences were discovered and submitted. In this way, our work provides a basis for understanding mechanisms of insecticide resistance and could greatly improve future studies of this pest at the molecular level.

However, it should be pointed out that although a large number of potentially interesting genes were obtained from the transcriptome data, most of them were partial sequences of specific genes and some of the unigenes were allelic variants or located on different part of the same gene. Due to short size or poor alignment, some sequences were excluded from analysis. In this way, when using this type of data to find genes of interest, particular attention should be paid to identifying each unigene to confirm that it is unique. To solve this problem, RACE technology is the preferred choice for future classification and obtaining the full length of these genes.

Because the functions of 22,349 of the unigenes assembled from the transcriptome remain unknown, we constructed DGE libraries for four different developmental stages to study the gene expression profiles of unigenes during the developmental process. We classified these differently-expressed genes using GO and KEGG categories to provide an overview of their functions and pathways. When comparing the gene expression profiles of E vs. L, the numbers of up-regulated and down-regulated genes were found to be similar. A similar situation was found in the L vs. P comparison, but the total number of genes was much lower than that in the E vs. L comparison. In P vs. A, most of the differently expressed genes were down-regulated in adults. Overall, 15,516 genes showed significant differences in expression during the transition from eggs to larvae, much more than in the development of larvae into pupae or of pupae into adults. This indicates that this might be the most complex stage of the *B. dorsalis* life cycle. Among the genes that differed in expression across different developmental stages, many were found to encode proteins of unknown function. This is in agreement with the results of DGE analysis of *N. lugens* and *B. tabaci* [23], [24]. Although many of the top regulated genes had no match in the NCBI database, differences in expression of genes encoding hormones and other factors related to development were observed. For example, the expression of the genes encoding juvenile hormone, juvenile hormone esterase, and ecdysis in *B. dorsalis* differed by developmental stage. This implies that the current understanding of the molecular mechanisms underlying insect development is still insufficient. The actual functions of more genes involved in development in *B. dorsalis* must be determined and made public. The lack of basic genetic information on *B. dorsalis* in GenBank and the limitations of the data size (2G) of the library containing all life stages (which we used as a reference) are the major obstacles to DGE data analysis and may cause the percentages of the reads mapping to reference to be skewed below the actual value when using DGE analysis. This not only reflects the necessity of transcriptome study to enrich the genetic information resources but also implies that more functional genes will be discovered as data size and capacity increase.

In conclusion, we sequenced the transcriptome of *B. dorsalis* and constructed a DGE library. These efforts revealed a large number of genes, both of known and unknown functions, greatly expanding the amount of genetic information available for this species and providing a profile of its developmental processes. This study is also a first step toward a better understanding of the functions of these genes and provides a broad and new vision of the future of research at the molecular level.

## Materials and Methods

### Insect samples

The laboratory colony of *B. dorsalis* was originally collected in 2008 from Haikou in Hainan Province, People's Republic of China. Adults were reared in cages and fed an artificial diet consisting of yeast powder, honey, sugar, ascorbic acid, and water. Females were induced to oviposit into pinpricked plastic tubes (50 mL) containing fresh orange pulp. Eggs were collected from these tubes. Third-instar larvae were transferred into a plastic basin containing sand until pupation. All specimens at all life stages were kept in a temperature-controlled room at 27±1°C, 70±5% relative humidity, and a photoperiod cycle of 14 h L/10 h D.

### RNA isolation

Total RNA was isolated from the following developmental stages: eggs collected within 24 h of oviposition; third-instar larvae; pupae; and newly-emerged adults (within five days of eclosion) in a 1∶1 female∶male ratio. For each developmental stage, approximately 8 mg of insects were homogenized with liquid nitrogen in a mortar. RNA was extracted using the RNeasy plus Micro Kit (Qiagen GmbH, Hilden, Germany) following the manufacturer's instructions. RNA was quantified by measuring the absorbance at 260 nm using a NanoVue UV-Vis spectrophotometer (GE Healthcare Bio-Science, Uppsala, Sweden). The purity of all RNA samples was assessed at an absorbance ratio of OD_260/280_ and OD_260/230_, and the integrity of RNA was confirmed by 1% agarose gel electrophoresis.

### Construction of the cDNA library and Illumina sequencing for transcriptome analysis

Briefly, 12 µg total RNA (a mixture of RNA from eggs, third-instar larvae, pupae, and adults at equal ratios) was used to construct a cDNA library. Poly (A) mRNA was purified from total RNA using oligo (dT) magnetic beads. It was then fragmented into small pieces by addition of fragmentation buffer. These short fragments served as templates to synthesize first-strand cDNA using random hexamer-primers. Second-strand cDNA was synthesized using buffer, dNTPs, RNaseH, and DNA polymerase I. Short fragments were purified using a QiaQuick PCR extraction kit. These fragments were washed with EB buffer for end reparation poly (A) addition and then ligated to sequencing adapters. Suitable fragments, as judged by agarose gel electrophoresis, were selected for use as templates for PCR amplification. The cDNA library was sequenced on Illumina HiSeq™ 2000 using paired-end technology in a single run (Figure S4A).

### Bioinformatics analysis

Transcriptome *de novo* assembly was carried out with the short-read assembly program SOAPdenovo (http://soap.genomics.org.cn/) as follows [40]: First, we used the overlap information from the short reads to construct high-coverage contigs without N. Then the reads were realigned onto contigs. The distance and relationships between these contigs were estimated with paired-end reads that enabled contig detection from the same transcript and from the distances between these contigs. Next we used SOAPdenovo to connect individual contigs into scaffolds using ambiguities (Ns) to represent unknown bases between adjacent contigs. Paired-end reads were used again to fill gaps in scaffolds to obtain sequences with the least Ns and could not be extended on either end, which were defined as unigenes. Finally, we screened our unigenes against protein databases like nr, Swiss-Prot, KEGG, and COG using BLASTX (E-value<10^−5^). The best hits were used to determine the sequence direction of the unigenes. When different databases gave conflicting results, we prioritized them in the following order: nr, then Swiss-Prot, then KEGG, then COG. When a unigene did not align with any of the entries in these databases, ESTScan was used to predict its coding regions and to determine its sequence direction (Figure S4B).

### Analysis of interested genes related to insecticide detoxification and target enzymes

Sequences encoding genes related to insecticide resistance, such as detoxification enzymes (GSTs, CarEs, and P450) and insecticide targets (AChE, GABA, sodium channel, and nAChRs), were identified by the BALST results against the nr database with a cut-off value of E<10^−5^. Sequences that returned redundant BLAST results or showed high homology with each other as determined by alignment results were eliminated as allelic variants or different parts of the same gene. MEGA 4.1 software was used to analyze the phylogenetic relationships between GSTs, CarEs, P450, and nAChRs genes with the related genes of *D. melanogaster* to make a prediction of their classification. The neighbor-joining method was used to create phylogenetic trees. Positions containing alignment gaps and missing data were eliminated with pairwise deletion. Bootstrap analysis of 1,000 replication trees was performed to evaluate the branch strength of each tree.

### Preparation and sequencing of the DGE library

RNA was extracted separately from eggs, third-instar larvae, pupae, and adults using RNeasy plus Micro Kits (Qiagen GmbH, Hilden, Germany) following the manufacturer's instruction, as described above. Approximately 10 µg RNA from specimens of each developmental stage was used to construct the DGE libraries. mRNA was treated as described in cDNA library construction. The required fragments were purified by agarose gel electrophoresis and enriched by PCR amplification. The library products were then ready for sequencing analysis via Illumina HiSeq 2000 using paired-end technology in a single run. Four libraries from each developmental stage were constructed (Figure S4A).

### Analysis and annotation of DGE tags

The original image data were converted into sequence data by base calling. Low-quality reads were omitted from data analysis. Low-quality reads were defined as (1) reads in which more than 50% of the bases had a quality value ≤5, (2) reads in which unknown reads were more than 10% per read, and (3) reads with adaptors. Clean reads were mapped to reference sequences (unigenes from the transcriptome data of four developmental stages was used as reference) using SOAPaligner/soap2 [41]. Mismatches of no more than two bases were allowed in the alignment. Gene expression levels were calculated using the RPKM method [42]. If there was more than one transcript for a given gene, the longest transcript was used to calculate its expression level and coverage. To identify differentially expressed genes between two samples, the false discovery rate (FDR) method was used to determine the threshold of *P*-value in multiple tests [43]. The significance of differences in gene expression was judged using a threshold FDR≤0.001 and an absolute value of log_2_Ratio ≥1. Then the genes that were expressed at different levels across samples were further annotated by GO function analysis and KEGG pathway analysis (Figure S4C).

### Validation of gene expression profile by semi-quantitative reverse transcription-PCR

RNA was extracted as described for the DGE library preparation and sequencing. A total of 2 µg of RNA from each developmental stage was reverse transcribed in a 20 µl reaction system using the PrimerScript™ RT Reagent Kit (Takara Biotechnology Dalian Co., Ltd., Dalian, China). Two genes that showed expression differences (either up-regulated or down-regulated) in three comparative groups (E vs. L, L vs. P, P vs. A) were randomly selected for validation. The α-tubulin gene (GU269902) of *B. dorsalis* was used as an internal control. Primer sequences are listed in Table 3. The 25 µL PCR reaction contained 1 µL cDNA template, 2 µL DNTP (Takara Biotechnology Dalian Co., Ltd., Dalian, China), 2.5 µL PCR buffer, 2.5 µL Mg^2+^, 1 µL of each primer, 15 µL water and 0.25 µL Taq polymerase (Takara Biotechnology Dalian Co., Ltd., Dalian, China). The PCR conditions for both genes were 95°C for 3 min, followed by 34 cycles of 94°C for 30 s, 60°C for 30 s, 72°C for 30 s, and a final extension at 72°C for 10 min. The PCR products of both genes were analyzed on a 1% agarose gel.

**Table 3:** Primers used in semi-quantitative RT-PCR.

Gene ID/Name	Primer sequences (forward)	Primer sequences (reverse)
40165	TTAGAGGAGCAACAGGTCAGTG	TCAACATCCAAAAGTTGCTGAG
43503	TGAAGGCAGCTGAATGTTTG	TCTTTGATGCGCAAACGTAG
45707	ACAAATCCAACCGAAAGCAG	CAACGCATTGAGATGCACTT
44480	GAAAACGCTGGATCAACTCC	CTTCCGCCTCTATTCCATGA
39779	ACGATAATGACATTGCTGTGCT	GGAAGGTGTACCACCATTTGTT
47697	TCGTGGAGTAGAAAATGAGCAA	GAAAGTTGGCGTTAATGTCCTC
α-TUB	CGCATTCATGGTTGATAACG	GGGCACCAAGTTAGTCTGGA

## Supporting Information

Figure S1 **Distribution of unigene lengths in the transcriptome of** ***Bactrocera dorsalis*** **.** The sizes of all unigenes were calculated.
(TIF)

Figure S2 **Evaluation of sequence quality for the four developmental stages of** ***Bactrocera dorsalis*** **.** E: eggs; L: larvae; P: pupae; A: adults.
(TIF)

Figure S3 **Distribution of gene coverage in each developmental stage of** ***Bactrocera dorsalis*** **.** E: eggs; L: larvae; P: pupae; A: adults.
(TIF)

Figure S4 **Experiment pipeline of RNA-Seq and bioinformatics analysis.**
(TIF)

Table S1 **Top hits obtained by BLASTX for the unigenes.**
(XLS)

Table S2 **KO annotation of unigenes.**
(XLS)

Table S3 **Sequence information of unigenes related to resistance.**
(XLS)

Table S4 **Top 10 up-regulated and down-regulated genes in E vs. L.**
(XLS)

Table S5 **Top 10 up-regulated and down-regulated genes in L vs. P.**
(XLS)

Table S6 **Top 10 up-regulated and down-regulated genes in P vs. A.**
(XLS)

Table S7 **GO function and KEGG pathway analysis results of E vs. L.**
(XLS)

Table S8 **GO function and KEGG pathway analysis results of L vs. P.**
(XLS)

Table S9 **GO function and KEGG pathway analysis results of P vs. A.**
(XLS)
