# Supplementary figures and images for: Transcriptome Analysis of the Oriental Fruit Fly (Bactrocera dorsalis)
Source: PLoS One. 2011 Dec 15;6(12):e29127. doi: 10.1371/journal.pone.0029127 (PMC3240649; doi:10.1371/journal.pone.0029127)

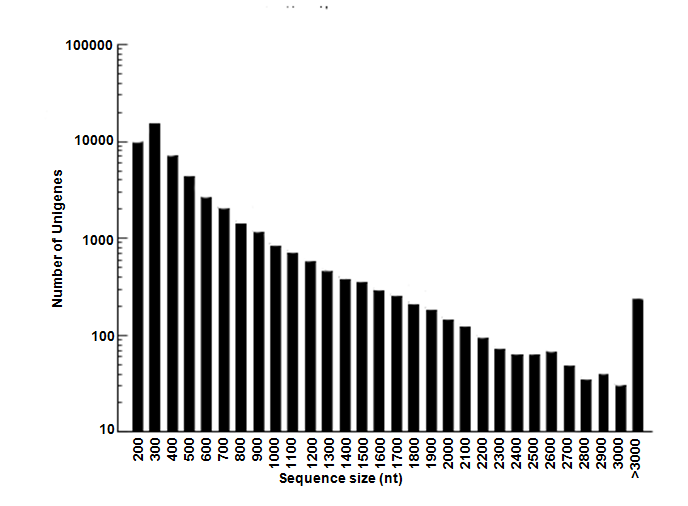

Supplement: Figure S1 — Distribution of unigene lengths in the transcriptome of Bactrocera dorsalis . The sizes of all unigenes were calculated. (TIF) [file pone.0029127.s001.tif]

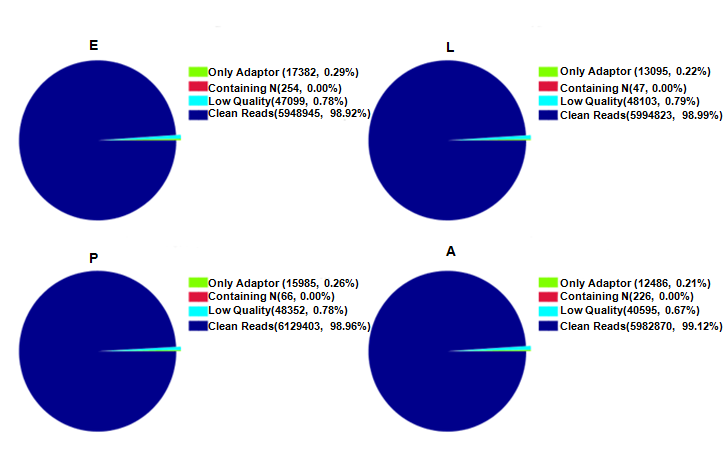

Supplement: Figure S2 — Evaluation of sequence quality for the four developmental stages of Bactrocera dorsalis . E: eggs; L: larvae; P: pupae; A: adults. (TIF) [file pone.0029127.s002.tif]

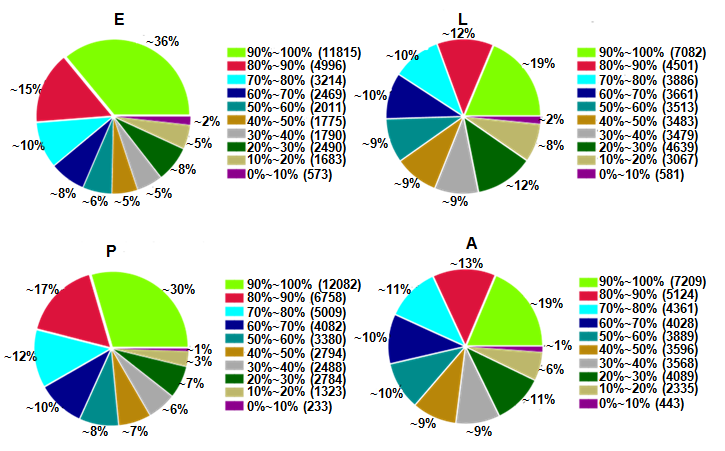

Supplement: Figure S3 — Distribution of gene coverage in each developmental stage of Bactrocera dorsalis . E: eggs; L: larvae; P: pupae; A: adults. (TIF) [file pone.0029127.s003.tif]

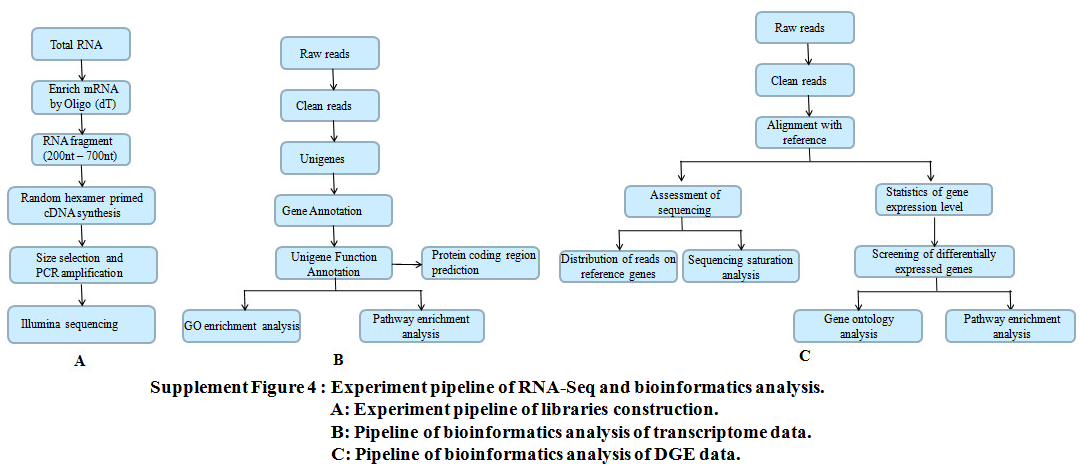

Supplement: Figure S4 — Experiment pipeline of RNA-Seq and bioinformatics analysis. (TIF) [file pone.0029127.s004.tif]
